# Measurement of and Trends in Unintended Birth in Bangladesh, 1983-2000

**DOI:** 10.3329/jhpn.v29i4.8457

**Published:** 2011-08

**Authors:** Jessica D. Gipson, Mian Bazle Hossain, Michael A. Koenig

**Affiliations:** ^1^Department of Community Health Sciences, UCLA School of Public Health, 650 Charles E. Young Drive South, CHS 46-071B, Los Angeles, CA 90095-1772, USA; ^2^School of Community Health and Policy Department of Public Health Analysis, Morgan State University, Baltimore, MD, USA,; ^3^Department of Population, Family and Reproductive Health, Johns Hopkins Bloomberg School of Public Health, Baltimore, MD, USA; ^*^Deceased

**Keywords:** Demographic transition, Fertility, Fertility decline, Bangladesh

## Abstract

Bangladesh has experienced a rapid decline in fertility in the past several decades, facilitated by proactive population policies, provision of contraceptives, and broader societal shifts, encouraging smaller families and use of contraceptive to achieve revised childbearing norms. This paper presents 18 years of data from the Sample Registration System, a demographic surveillance system operated by the Maternal and Child Health–Family Planning Extension Project in six study areas in Bangladesh. Prospective measurements of women's fertility preferences were used for classifying nearly 25,000 birth outcomes from 1983 to 2000 as intended, unintended, or ‘up to God/Allah’. Over the 18-year period, the level of unintended births varied from 22% to 38%, with the lowest levels in the mid-1990s. Fatalistic responses declined significantly from 25% in the mid-1980s to 1% by the late 1990s. Results of the comparison of two geographic areas of Bangladesh indicate differential declines in the levels of unintended pregnancies over the study period. Pros-pective measurements of unintended pregnancies were 2-3 times the magnitude indicated by retrospective estimates of unwanted births from the demographic and health surveys conducted during the study period. This unique dataset provides a rare opportunity to visualize the vast changes in fertility preferences and unintended births in Bangladesh from 1983 to 2000. Significant declines in fatalistic responses reflect broader social changes that occurred in Bangladesh to facilitate the fertility decline and contraceptive uptake. The drastic differences between prospective and retrospective measurements of fertility preferences highlight the importance of considering the strengths and limitations of each method when attempting to estimate the true level of unintended pregnancies and births in a population.

## INTRODUCTION

Bangladesh is touted as a success story of demographic transition. In the 1970s, the total fertility rate (TFR) was just under seven births per woman, with 8% of women using contraception (1). By the 1999 Bangladesh Demographic and Health Survey (BDHS), the total fertility rate had dropped to 3.3, and 54% of currently-married women reported using contraception, with further progress documented by the 2007 BDHS (2,3). There are several factors integral to this fertility decline—the proactive policies of the national government with respect to the promotion of family planning and the legal provision of menstrual regulation (4,5). Menstrual regulation is the use of manual vacuum aspiration to restore menstruation, even without the confirmation of pregnancy status. The fertility decline in Bangladesh cannot only be attributed to family-planning programmes and policies but also to the broader social and psychological shift in family-size norms as indicated by in-depth work conducted in Bangladesh during this time (6,7).

Bongaarts has described the changes in the proximate determinants of fertility as nations undergo a demographic transition and the subsequent impact on the levels of unintended pregnancies (8). Individuals and couples first perceive a greater benefit of having smaller families. However, as a smaller family-size becomes the ideal, it is likely that there will be a gap between fertility preferences and the enactment of these preferences through fertility control. This gap equates to, at least initially, a higher level in unintended pregnancies. However, as couples and societies embrace a smaller family-size norm and policies and programmes support these revised preferences through the provision of quality services, the use of contraceptives will, ideally, begin to match fertility preferences, and unintended pregnancies will decline.

To assess these broader societal changes, it is important to measure fertility preferences and their impact on actual fertility. Most work examining fertility preferences is derived from demographic and health survey data, which assess fertility preferences through retrospective assessments of fertili-ty preferences for births occurring within the past 3-5 years. Retrospective assessments are subject to rationalization bias as women are much less likely to characterize a pregnancy as ‘unwanted’ after the birth of the child (9,10). Prospective fertility preferences have been shown to be predictive of subsequent fertility; however, these assessments also have their limitations. For example, preferences may change over time and in response to intervening circumstances, e.g. death of a child and change in economic situation of family (11-13).

In addition to concerns regarding the timing of measurements, existing studies also note difficulties in ascertaining valid measurements of fertility preferences due to differences in the way that fertili-ty is conceptualized by individuals and is believed to be under one's control. Studies across cultural settings document the existence of fatalistic or ‘up to God’ responses when asked about the desired number of children. Non-numeric responses may be more likely in populations in which fertility is not considered to be within ‘calculus of conscious choice’ of individuals (14), in populations where family-size is not conceptualized in terms of a numeric value (15), or in contexts in which there is high uncertainty concerning child survival (16,17). Educational attainment and exposure to a more ‘modern’ worldview have also been hypothesized to negatively influence the occurrence of non-numeric responses (18,19).

To assess fertility preferences and subsequent fertility, we used a unique, longitudinal dataset from the Maternal and Child Health–Family Planning (MCH-FP) Extension project sites in Bangladesh from 1982 to 2000. Using this powerful dataset, we were able to match prospective fertility preferences of women with nearly 20,000 subsequent birth outcomes and to assess the levels of and trends in unintended births over nearly two decades across six different areas of Bangladesh.

## MATERIALS AND METHODS

### Study sites

In 1982, the International Centre for Diarrhoeal Disease Research, Bangladesh (ICDDR,B) established the MCH-FP Extension Project and the Sample Registration System (SRS) in Jessore (southwest) and Sirajganj (north-central) areas of Bangladesh (20). The MCH-FP Extension Project areas comprised intervention and comparison sites to test the effectiveness of public-sector MCH-FP projects. In Jessore district, Abhoynagar was the intervention site, with the adjacent upazilas (administrative unit comprising approximately 300,000 people) of Fultala, Bagherpara, and Keshobpur serving as the control areas. The Sirajganj community served as the intervention site in the north-central project area, with Gopalpur as the control community. All the study communities are located in rural areas of Bangladesh; however, there are economic and social differences between the two study areas. The Jessore site is characterized by greater economic development and infrastructure compared to the Sirajganj site (13).

### Data

The SRS was a quarterly surveillance system that registered demographic and health events in approximately 8,000 households across the study areas (20). In addition to the quarterly collection of demographic data, periodic knowledge, attitude, and practice (KAP) surveys were administered at an interval of 3-5 years. In each KAP survey in 1982, 1985, 1990, 1993, and 1998, currently-married female participants were asked about their fertility preferences. If pregnant, female respondents were asked: “With the present pregnancy outcome, how many additional children do you want?” If not pregnant at the time of the survey, female respondents were asked: “How many (additional) children do you want?”

The dataset used in this analysis was constructed by merging the cross-sectional data from the five KAP surveys with all the births registered during 1983-2000 in the SRS longitudinal surveillance database. A set of algorithms was established to link each birth to a prior measurement of fertility preference: (a) women with pregnancies or births without a prior measurement of fertility preference were excluded (Table); (b) births were linked to measurements of fertility preference from the closest, previous survey, up to two prior surveys; and (c) if multiple pregnancies occurred between KAP surveys, it was assumed that prior fertility preferences applied to all pregnancies.

A member of the research team reviewed and hand-coded data relating to each study participant. The lead author cross-checked every tenth case. To decrease the number of excluded cases, the research team searched over 25,000 hard-copy files.

## RESULTS

The table presents the sociodemographic characteristics of the included versus excluded cases. The large majority (74%) of the excluded cases were women who had migrated into the study area upon marriage, for whom the SRS had registered a birth but without a prior measurement of fertili-ty preference gathered through the periodic KAP surveys. Aligning with this profile, the table shows that the excluded cases were younger, lower pari-ty, and more educated [Higher levels of education among younger women are not surprising, given the increased governmental support in the 1990s for enrollment in primary schools, especially among girls (21)].

**Table 1.** Comparison of included and excluded cases

Characteristics	Included	Excluded
	(n=19,934)	(n=5,113)
Current age of mother (mean)*	32.9	26.6
No. of children ever born*	3.6	1.4
% of any maternal education*	35.6	44.8
% of any paternal education	47.2	47.0
% of Hindus	11.0	12.0

*p<0.05

**Fig. 1.**
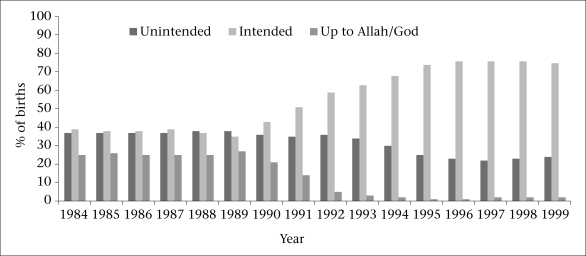
Reported fertility preferences across 6 study areas, 1984-1999 (n=19,934)

Figure 1 shows the levels of births labelled as intended, unintended, and up to God/Allah that occurred from 1983 to 2000 [Smoothed estimates are displayed; thus, only years 1984-1999 are depicted]. Over the 18-year period, unintended pregnancy levels varied from 22% to 38%, with the lowest levels in the mid-1990s. Fatalistic responses declined significantly (p<0.001) over the study period from 25% in the mid-1980s to 1% in the mid-1990s.

**Fig. 2.**
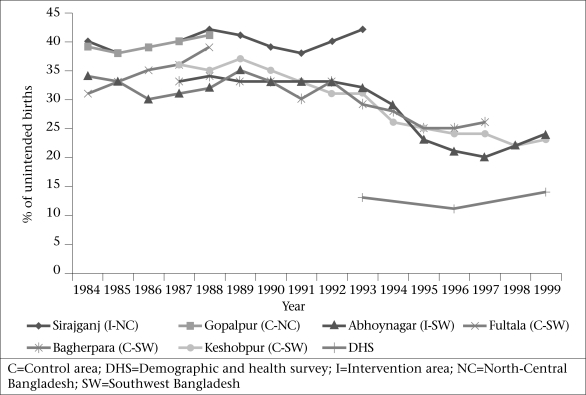
Trends in unintended births in 6 study sites (1984-1999) and DHS estimates

Figure 2 compares the level of unintended births that occurred across the six sites throughout the study period. Of note is the difference in levels of unintended pregnancies between the north-central Sirajganj sites and the southwestern Jessore study sites. Approximately 40% of births were unintended in the Sirajganj sites during 1984-1993, compared to 30-35% in the Jessore sites. Although included as a Jessore comparison site for a shorter span of time, the Fultala site had the highest levels of unintended pregnancies of the southwestern sites. The trends from the southwestern study areas also indicate a downward trend in the levels of unintended births. Levels of unintended pregnancies in Abhoynagar, the southwestern intervention site, declined from 34% in 1984 to 20% in 1997; however, by the last point of data-collection in 1999, levels of unintended pregnancies had risen slightly to 24%.

Figure 2 also depicts the stark differences in unintended births as measured by prospective fertility preferences compared to the retrospective DHS estimates of unwanted births from 1993, 1996, and 1999. In 1993, 29-42% of births were prospectively classified as unintended (or unwanted, since these pregnancies occurred to women who indicated they did not want *any more* children) compared to 13% of births that were retrospectively classified by the DHS as unwanted. In 1996 and 1999, the discrepancies between MCH-FP and DHS data are similarly large: 21-25% versus 11% of births in 1996 and 23-24% versus 14% of births in 1999 were classified as unwanted via prospective and retrospective measurements respectively.

## DISCUSSION

This unique, longitudinal dataset provides a rare opportunity to assess the prevalence of and trends in unintended births over an 18-year study period across six areas in Bangladesh, a setting characterized by progressive family-planning policies and programmes and rapid fertility decline. These analyses yield three key insights into the measurement of and trends in unintended pregnancies.

First, these data clearly illustrate the significant decline in fatalistic or ‘up to God’ responses throughout the study period, documenting the broader transitions in childbearing norms that facilitated drastic changes in fertility rates and contraceptive prevalence during this time in Bangladesh. As first noted by Coale, the shift away from fatalistic responses over the study period is likely reflecting the notion of ‘calculus of conscious choice’ in which individuals and couples begin to identify fertili-ty as something that is within one's control and can be regulated (14). Numerous quantitative and qualitative studies from Bangladesh help explain and contextualize this shift in the perceived control over fertility and the use of contraception to achieve desired preferences, pointing to the role of increased educational and microcredit opportunities, decreased infant mortality rates, widespread acceptance and provision of modern family planning through the national family-planning programme, and the changing roles of women as promoters and acceptors of contraception (7,22-27).

Second, data from three of the six study areas mirror the trends in unintended pregnancies hypothesized by Bongaarts as individuals and couples first develop the idea of, then attempt to achieve, smaller family-sizes (8). Data (1983-2000) from the Jessore study area indicate an increase in the level of unintended pregnancies from the early to late 1980s, followed by a steady decline throughout the 1990s. This trend, however, was not indicated in the earlier data from the less-developed Sirajganj areas. Although the Sirajganj study areas were phased out in the early 1990s, data from the 1999 BDHS indicated persistent differences between Khulna division (where Jessore is located) and Rajshahi division (where Sirajganj is located), with the Khulna area characterized by higher contraceptive prevalence and lower fertility rates than Rajshahi division (28). Although data from only two geographic regions are presented here, these two areas reflect the broader heterogeneity within Bangladesh with respect to fertility preferences (29).

Third, these data provide a rare perspective by matching prospective assessments of fertility preferences with subsequent birth outcomes. Although these measurements have their respective limitations, results of the comparisons of prospective versus retrospective assessments indicate that retrospective assessments likely underestimate the true level of unintended pregnancies/births (10,30). In this analysis, women were asked to report about their desired future childbearing. Although some women may have experienced intervening circumstances or changes in their fertility preferences, births occurring to women who stated a desire not to have any (more) children in the future likely approximate births considered to be ‘unwanted’, a category used in the DHS to des-cribe births that occurred when another birth was ‘not wanted at all’. When comparing the proportion of women who reported an unwanted pregnancy, these prospective estimates are 2-3 times the levels of unwanted births recorded in the national DHS estimates.

### Limitations

A few limitations of the study should be mentioned. The measures of fertility preference that were used across the MCH-FP surveys were usually trichotomous (want more; do not want more; up to God). Results of more recent studies have pointed to the utility of a scaled measure to better represent the nuances of fertility preferences (12,30). In addition, across the time period, these preferences were consistently measured for women only. Results of a separate analysis of data from the 1998-2003 MCH-FP data indicated the importance of considering fertility preferences of husbands for subsequent reproductive outcomes; however, these measurements were not available for the duration of the study (31,32). This dataset includes only livebirth outcomes, underestimating the true level of unintended pregnancies in the population. In the analysis of 1998-2003 data, 11% of all pregnancies were terminated via menstrual regulation over the five-year study period (31). Lastly, due to the inability to classify each birth that occurred between the KAP surveys, it was assumed that prior fertility preferences applied to all pregnancies that occurred within the gap between the KAP surveys. However, this assumption would likely result in a more conservative estimate of unintended pregnancies since the characterization of a pregnancy or birth as being unwanted increases with parity.

### Conclusions

This unique dataset provides a rare opportunity to visualize the vast changes in fertility preferences and unintended births in Bangladesh from 1983 to 2000. The significant declines in fatalistic responses reflect broader social changes that were occurring in Bangladesh to facilitate the fertility decline and contraceptive uptake. The drastic differences between pros-pective and retrospective measurements of fertility preferences highlight the importance of considering the strengths and limitations of each method when attempting to estimate the true level of unintended pregnancies and births in a population.

## ACKNOWLEDGEMENTS

The authors acknowledge funding from the United States Agency for International Development (USAID), Dhaka office (GHS-A-00-03-00019-00) for this project. They thank several team members from ICDDR,B, who were integral to the collection, compilation, and analysis of the MCH-FP Project data, including Khorshed Mozumder, Ahsan Kabir, Mizanur Rahman, Rezaul Kabir, Siddiqur Rahman, Subash Das, and Carel van Mels.
